# Masculinized Second-to-Fourth Digit Ratio (2D:4D Ratio) Is Associated With Lower Cortisol Response in Infant Female Rhesus Monkeys (*Macaca mulatta*)

**DOI:** 10.3389/fnbeh.2020.00094

**Published:** 2020-09-03

**Authors:** Elizabeth K. Wood, Parker Jarman, Elysha Cash, Alexander Baxter, John P. Capitanio, J. Dee Higley

**Affiliations:** ^1^Department of Psychology, Brigham Young University, Provo, UT, United States; ^2^Department of Psychology, University of California, Davis, Davis, CA, United States; ^3^California National Primate Research Center (CNPRC), Davis, CA, United States

**Keywords:** 2D:4D ratio, cortisol, HPA axis, prenatal androgen exposure, rhesus monkeys, stress

## Abstract

The second-to-fourth digit ratio (2D:4D ratio) is considered a postnatal proxy measure for the degree of prenatal androgen exposure (PAE), which is the primary factor responsible for masculinizing the brain of a developing fetus. Some studies suggest that the organizational effects of PAE may extend to the hypothalamic-pituitary-adrenal (HPA) axis response to stress. This study investigates the relationship between 2D:4D ratio and HPA axis functioning using a rhesus monkey (*Macaca mulatta*) model. Subjects were *N* = 268 (180 females, 88 males) rhesus monkey infants (3–4 months of age). Plasma cortisol concentrations were assayed from two blood samples obtained during a 25-h experimental social separation stressor at 2- and 7-h post-separation. Subjects’ 2D:4D ratio was measured later in life (*M*_age_ = 6.70 years). It was hypothesized that infant rhesus monkeys that exhibited a more masculine-like 2D:4D ratio would show lower levels of circulating cortisol after a social separation and relocation stressor. The results showed that there was a sex difference in the left-hand 2D:4D ratio. The results also showed that there was an overall sex difference in cortisol concentrations and that female, but not male, monkeys that exhibited a more masculine-like right- and left-hand 2D:4D ratio exhibited lower mean stress-induced cortisol concentrations early in life. These findings suggest that higher levels of prenatal androgens in females, as measured by 2D:4D ratio, may be related to an attenuated HPA axis stress-response, as measured by plasma cortisol levels. To the extent that these findings generalize to humans, they suggest that the organizational effects of PAE extend to the infant HPA axis, modulating the HPA axis response, particularly in females.

## Introduction

Prenatal androgen exposure (PAE) is thought to be the main source of morphological and central nervous system masculinization, and, consequently, is responsible for many of the phenotypic behavioral differences observed between males and females (Phoenix et al., 1959; Hughes, 2001; Thornton et al., 2009). During gestation, androgens bathe the brain, initiating enzymatic cascades that masculinize the developing fetus (Hughes, 2001) through a variety of epigenetic mechanisms (Gegenhuber and Tollkuhn, 2019). The degree of PAE varies between the sexes (males typically have a higher degree of PAE; Wilson et al., 1981), but there are also wide individual differences within each of the sexes. Studies show that variation in PAE contributes to stable individual differences in brain function and behavior (Hines et al., 2016; Spencer et al., 2017; Del Giudice et al., 2018). Second-to-forth digit ratio (2D:4D ratio) is associated with sex differences in behavior, for example, one well-replicated line of research shows the same-sex attraction in women is associated with a more masculinized 2D:4D ratio (Williams et al., 2000; Kraemer et al., 2006; Watts et al., 2018), although the inverse relationship is not always found in men (Williams et al., 2000; Voracek et al., 2005). Thus, PAE plays a complex and important modulating role in the development of typical sex differences through its organizational effect on the brain. Studies also show that PAE is implicated in the development of several psychopathological disorders with extant sex differences in incidence rates, such as autism spectrum disorder (Cherskov et al., 2018), schizophrenia (Paipa et al., 2018), attention deficit hyperactive disorder (Martel et al., 2008) and, particularly relevant to this study, anxiety disorders (de Bruin et al., 2006).

Given its organizational effects on the brain and periphery, PAE may modulate the hypothalamic-pituitary-adrenal (HPA) axis. Numerous studies suggest that there are sex differences in the functioning of the HPA axis (for a review, see Handa et al., 1994). For example, using the same paradigm described in the present study, Capitanio et al. (2005) assessed sex differences in HPA axis response. Briefly, this paradigm consists of a 25-h stress-inducing social separation of infant rhesus monkeys from their mothers. During this period, infant subjects are assessed on a variety of biobehavioral metrics, including undergoing blood sampling at 2-h and 7-h post-separation, respectively. Obtained plasma cortisol is then assayed for cortisol concentrations using radioimmunoassay (for a detailed description of this methodology, see Capitanio et al., 2005). Using this paradigm, Capitanio et al. (2005) showed that male rhesus monkey infants exhibit lower plasma cortisol and are less responsive to dexamethasone and adrenocorticotropic hormone (ACTH) during an experimental social separation and relocation stressor, when compared to females, replicating plasma cortisol and ACTH findings in human subjects (Kudielka et al., 2004). Studies also suggest that the plasma cortisol levels of females may be more sensitive to other variables that affect the response of the HPA axis to stress (Uhart et al., 2006), with research suggesting that the greater prevalence of women with depression, when compared to men, may be related to the tendency of females to show an elevated HPA axis response to stress when compared to males (for a review, see Bale and Epperson, 2015). Given these tendencies, more PAE may lead to a more masculinized HPA axis in males, when compared to females, although few studies have assessed this possibility. As stress reactivity is a complex and emergent phenomenon, influenced by both the organizational and activational effects of testosterone, there is a greater need to understand the individual contributions of PAE in an organizational capacity.

While direct measures of PAE can be made by extracting amniotic fluid (Spencer et al., 2017; Beking et al., 2018; Wang et al., 2019), there are decided limitations to this methodology. For example, the composition of extracted amniotic fluid represents androgen levels at a single time point, which may not capture the day-to-day variability of PAE or the sustained effect of higher mean levels of PAE. While some have attempted to measure PAE by other means, such as assaying venous or arterial umbilical cord blood, these data often do not correlate with concomitant androgen levels in the amniotic fluid (van de Beek et al., 2004). Moreover, none of these methods, including assaying amniotic fluid for androgen levels using a single point in time, provide a long-term chronic pooled estimate of PAE exposure. Given the difficulty in collecting representative samples across pregnancy, many researchers have opted for proxy measurements of PAE, such as the 2D:4D ratio, first proposed by Manning et al. (1998), and widely used by others following the initial publication (Hönekopp et al., 2007). While somewhat controversial, the 2D:4D ratio has been widely used to investigate biological influences on gender differences. Comparisons of 2D:4D ratios and phenotype have not always shown clear sex differences and its use as a proxy to demonstrate prenatal contributions to gender differences are sometimes inconsistent, with recent commentaries (see Swift-Gallant et al., 2020) suggesting myriad reasons including small sample sizes, variation in behavior, that 2D:4D ratio is intended as an imperfect proxy for PAE, and that PAE is only partially responsible for variation in 2D:4D ratio. Indeed, while 2D:4D ratio provides a useful measure of chronic pooled PAE, other influences on 2D:4D ratio, such as genetic influences, cannot be ruled out, nor are they mutually exclusive. The bulk of the studies suggest that the 2D:4D ratio is sexually-dimorphic in humans (for a review, see Manning, 2011) and non-human primates (Nelson and Shultz, 2010), though the direction of the dimorphism may be species-specific, with male rhesus monkeys typically exhibiting a higher 2D:4D ratio, while female humans typically exhibit a higher 2D:4D ratio (Baxter et al., 2018).

PAE is thought to be, at least in part, responsible for 2D:4D ratio, with sex differences in 2D:4D ratio already apparent prenatally (Galis et al., 2010). For example, a recent study showed a significant relationship between urinary testosterone levels in pregnant female monkeys and subsequent 2D:4D ratio of offspring (Baxter et al., 2019). Studies in nonhuman primates also show that experimentally increasing circulating prenatal androgens, thus increasing PAE, masculinizes 2D:4D ratios (Abbott et al., 2012). Similarly, studies investigating congenital adrenal hyperplasia, a condition that leads to abnormally high PAE, show that female humans with this condition exhibit a more masculinized 2D:4D ratio (Brown et al., 2002; Rivas et al., 2014). Studies also suggest that the effects of PAE extend to behavior and temperament. For example, one study showed that a feminized 2D:4D ratio in women (but not men) is associated with increased temperamental harm avoidance (Jeon et al., 2016), which may be related to the organizational effects of PAE on the HPA axis. Another study in young adult females showed that experimental administration of testosterone led to reductions of cognitive empathy, and this testosterone-induced low empathy was related to PAE, as measured by 2D:4D ratio (van Honk et al., 2011). Such 2D:4D relationships with harm avoidance and empathy suggest that prenatal organizational effects likely modulate gender differences in mood and may explain, at least in part, male-female differences in the risk for mood disorders.

Researchers have noted the utility of rhesus monkeys (*Macaca mulatta*) for studying organizational effects of PAE on the brain (Thornton et al., 2009; Baxter et al., 2018), due to their genetic (Gibbs et al., 2007), temperamental (Weinstein and Capitanio, 2008), and social (Capitanio, 1985) similarities to humans. Particularly relevant to this study, the rhesus monkey response to stress has been widely studied and is well-characterized (see Sanchez, 2006). One important strength of utilizing a rhesus monkey, rather than a human, model to investigate the relationship between 2D:4D ratio and development is that the rhesus monkey environment is closely controlled, eliminating extraneous variables that may impact human development (for example, socioeconomic status or race; Henry et al., 2019). This, plus the relative ease of obtaining direct measurements, increases the ability to assess potential causal mechanisms with a higher degree of certainty. The present study investigated the relationship between PAE, as measured by 2D:4D ratio, and early-life HPA axis response to stress, as measured by circulating cortisol concentrations in infant male and female rhesus monkeys during an ecologically-meaningful, well-validated stressor. The purpose of this study is to investigate the organizational effects of PAE on the HPA axis. To the extent that women are at greater risk for mood disorders that are HPA-axis-related (Rainville and Hodes, 2019), investigating whether higher PAE in females has a protective effect while lower PAE in males is a risk factor for dysregulated HPA axis function may provide important information concerning both the etiology of sex differences in the HPA axis and the organizational effects of PAE on subsequent risk for anxiety and depression. Based on earlier findings showing sex differences in plasma cortisol response to a social stressor (Capitanio et al., 2005), it is hypothesized that infant rhesus monkey females will have a greater cortisol response to social separation from their mother and their social group when compared to males. Given earlier findings of a sex difference in 2D:4D ratio of rhesus monkeys (Baxter et al., 2018), it is hypothesized that there will be a sex difference in 2D:4D ratio, such that male rhesus monkeys will exhibit a higher 2D:4D ratio pattern, when compared to female rhesus monkeys. Furthermore, given findings suggesting the relationship between female-typical 2D:4D ratio and personality/temperament in women (Jeon et al., 2016) and men (Evardone and Alexander, 2009) and other studies showing that females tend to exhibit greater sensitivity to environmental moderators of the HPA axis (Barr et al., 2004; Uhart et al., 2006), it is hypothesized that females with a more male-typical 2D:4D ratio will exhibit lower plasma cortisol concentrations in response to stress when compared to other females, while males with a more female-typical 2D:4D ratio will exhibit higher cortisol concentrations in response to stress when compared to other males.

## Materials and Methods

Subjects were *N* = 268 rhesus monkeys (180 females, 88 males) housed at the California National Primate Research Center (CNPRC) in Davis, California in outdoor, 0.2-hectare field cages. Subjects lived in large social groups (approximately 60–100 animals of all age and sex classes), which is about the same size as typical rhesus monkey groups, in conditions approximating the natural social composition (matrilineally organized extended-family groups with multiple adult males, infants, and juveniles). This study was carried out following the recommendations of the Guide for the Care and Use of Laboratory Animals, National Institutes of Health, and with the guidelines established by the California National Primate Research Center (CNPRC). All procedures were reviewed and approved by the Animal Care and Use Committee of the University of California-Davis.

### Cortisol Sampling

Cortisol samples were obtained when the subjects were infants, during standardized experimental testing outlined by Capitanio et al. (2005). Briefly, at 3–4 months of age, infants were separated from their mothers and their larger social groups and underwent a standardized, 25-h biobehavioral assessment, in which they participated in a wide variety of biobehavioral tests. As part of the testing battery, blood samples were obtained *via* femoral venipuncture at 2-h following separation (11:00 h) and again approximately 5-h later (16:00 h). All blood samples were drawn using unheparinized syringes and immediately transferred to EDTA tubes. The samples were centrifuged at 4°C at 1,277 *g* for 10 min. Plasma was pipetted into tubes and stored at −80°C until they were assayed for cortisol concentrations. All cortisol data were collected between 2001–2016. The samples collected before 2014 (*n* = 151), were assayed using a commercial radioimmunoassay kit (Siemens Medical Solutions Diagnostics, Los Angeles, CA, USA). Samples collected after 2014 (*n* = 117), were assayed using a quantitative competitive immunoassay (Siemens Healthcare Diagnostics, Tarrytown, New York, NY, USA). For a description of each assaying procedure, see Vandeleest et al. (2019). There were no significant differences in the mean cortisol concentrations assayed using the two methods (*t*_(264)_ = 0.56, *p* = 0.580). As preliminary analyses showed that the two cortisol samples were significantly and positively correlated across time points (*r* = 0.74, *p* < 0.0001), mean cortisol concentrations were used in analyses. To account for any variance due to cohort year, cortisol concentrations were statistically standardized across cohort years and the resulting standardized values were used in all further analyses.

### Digit Ratio Measurements

Digits were measured between 1–17 years after cortisol sampling during routine biannual health examinations. As part of these routine examinations, subjects were sedated with ketamine (15 mg/kg, intramuscular). Two technicians measured the fingers by working together, using the same procedure described in Baxter et al. (2018). Briefly, monkeys were laid in a recumbent position on a table, and, to increase accuracy, the first technician used a wooden craft stick to depress and restrain the monkeys’ palms and fingers flat against the table. A second technician measured the restrained fingers using a digital caliper, measuring from the finger-crease most proximate to the palm to the most distal point of the finger, following guidelines from Manning (2011). Using a caliper to directly measure fingers may yield more accurate and reliable measures than indirect measurements from photos or scans (Fink and Manning, 2018). Subjects’ second and fourth fingers of their right and left hands were measured at least twice, until at least two measurements were obtained within ± 1.5 mm, and the average length of the finger was calculated by averaging the two closest measurements. Subjects’ 2D:4D ratios were calculated by dividing the average length of the second finger by the average length of the fourth finger for each hand. All digit ratio data were collected between 2016–2018 (inter-rater reliability >0.90). Preliminary analyses showed that right-hand 2D:4D ratio and left-hand 2D:4D ratio were significantly and positively correlated (*r* = 0.27, *p* < 0.001). Right- and left-hand 2D:4D ratios were statistically standardized across cohort years and the resulting standardized right- and left-hand 2D:4D ratio values were used in all further analyses. For ease of interpretation, all figures depict unstandardized values.

### Data Analysis

As an earlier study showed a significant relationship between 3–4 month infant monkeys’ cortisol concentrations and age (Capitanio et al., 2005), infant age (days old) at the time of cortisol sampling was controlled in all analyses. ANOVAs were used to test for sex differences in 2D:4D ratio, with sex as the independent variable and left- or right-hand 2D:4D ratio as the dependent variable. An ANOVA was also used to test for sex differences in plasma cortisol, with sex as the independent variable, mean plasma cortisol concentrations as the dependent variable, and infant age entered as a covariate.

Multiple regression was used to test the relationship between cortisol and 2D:4D ratio, with right- or left-hand 2D:4D ratio and infant age as the independent variables and mean plasma cortisol concentrations as the dependent variable. Because preliminary analyses showed sex differences in cortisol concentrations (*p* < 0.0001), the multiple regression analyses were performed separately for males and females. All analyses were performed using SPSS, version 25.

## Results

**Figure 1:**
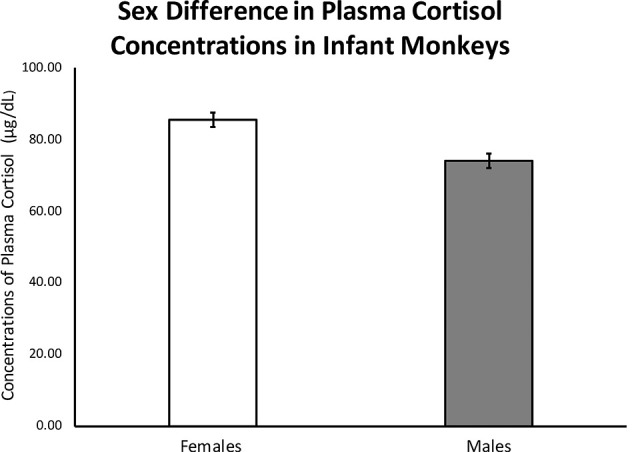
Results from an ANOVA with sex as the independent variable and infant stress-response cortisol concentrations as the dependent variable and infant age entered as a covariate showed a significant effect of sex on stress-induced cortisol concentrations in 3-to-4-month-old infants (*F*_(1,263)_ = 18.20, *p* < 0.0001). Error bars represent standard error of the mean.

As hypothesized, results from ANOVA showed a significant sex difference in mean cortisol concentrations between males (*M* = 69.17 ± 1.93) and females (*M* = 81.80 ± 1.84); *F*_(1,263)_ = 18.20, *p* < 0.0001; see Figure 1). There was also a significant sex difference in the left-hand 2D:4D ratio (*F*_(1,248)_ = 6.837, *p* = 0.009), with males exhibiting a higher left-hand 2D:4D ratio (0.81 ± 0.003), when compared to females (0.80 ± 0.002). There was not a detectable sex difference in the right-hand 2D:4D ratio (*p* = 0.22).

### Females

**Figure 2:**
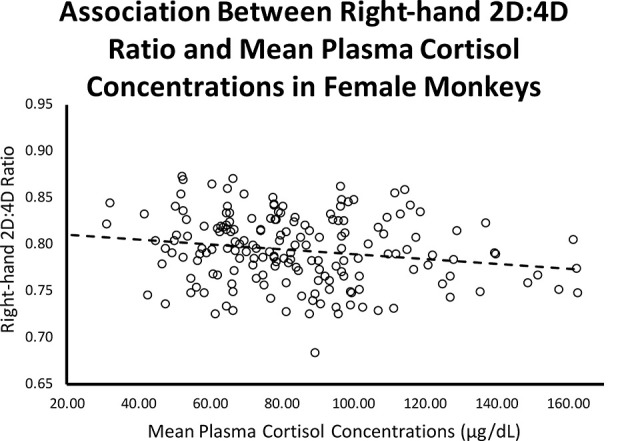
Controlling for infant age, there was a significant (*β* = −0.204, *p* = 0.007; Overall model: *R* = 0.203, *F*_(2,173)_ = 3.70, *p* = 0.027) negative relationship between right-hand 2D:4D ratio and stress-induced plasma cortisol concentrations for female rhesus monkeys.

Controlling for infant age at cortisol sampling, results from a multiple regression analysis showed a significant negative relationship between right-hand 2D:4D ratio and mean plasma cortisol concentrations for females (*β* = −0.204, *p* = 0.007; Overall model: *R* = 0.203, *F*_(2,173)_ = 3.70, *p* = 0.027; see Figure 2).

**Figure 3:**
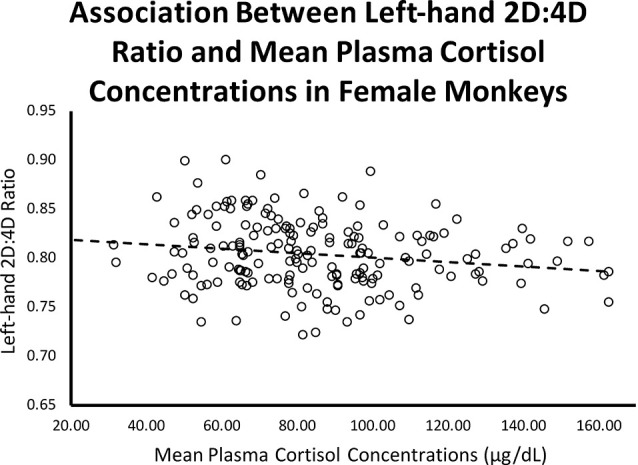
Controlling for infant age, there was a significant (*β* = −0.199, *p* = 0.009; Overall model: *R* = 0.198, *F*_(2,173)_ = 3.54, *p* = 0.031) negative relationship between left-hand 2D:4D ratio and stress-induced plasma cortisol concentrations for female rhesus monkeys.

Controlling for infant age at cortisol sampling, results from a multiple regression analysis showed a significant negative relationship between left-hand 2D:4D ratio and mean plasma cortisol concentrations for females (*β* = −0.199, *p* = 0.009; Overall model: *R* = 0.198, *F*_(2,173)_ = 3.54, *p* = 0.031; see Figure 3).

### Males

The relationship between right-hand 2D:4D ratio and infant cortisol concentrations was not significant for males (*β* = 0.023, *p* = 0.837; Overall model: *R* = 0.076, *F*_(2,82)_ = 0.24, *p* = 0.789), nor was the relationship between left-hand 2D:4D ratio and infant cortisol concentrations (*β* = 0.010, *p* = 0.929; Overall model: *R* = 0.073, *F*_(2,82)_ = 0.22, *p* = 0.803).

## Discussion

Partial support was found for the hypotheses: There was a relationship between circulating stress-response plasma cortisol and 2D:4D ratio in females, with females that possessed a more male-typical 2D:4D ratio exhibiting lower plasma cortisol concentrations as infants during a mother-infant social separation stressor. To illustrate this point, 61% of females with 2D:4D ratios that were at or above the male 2D:4D ratio average had cortisol concentrations that were comparable to males. For the males, however, there was no relationship between 2D:4D ratio and infant plasma cortisol concentrations. To the extent that the 2D:4D ratio is a biomarker for the degree of PAE, these results suggest that PAE has a prenatal organizational effect on the HPA axis, which appears to attenuate the stress response of the HPA axis in female rhesus monkeys. To our knowledge, this is the first report of a relationship between PAE and stress-induced plasma cortisol levels.

One explanation for the finding that there is a relationship between infant plasma cortisol concentrations and 2D:4D ratio in females, but not males, is that high levels of PAE may lead to organizational changes that masculinize the HPA axis, leading to a more masculinized response in females. Specifically, infant females that were likely exposed to relatively higher levels of prenatal androgens (as indicated by their 2D:4D ratio) showed an attenuated, male-like cortisol response to a social separation stressor, at least at the level of the HPA axis. This interpretation is corroborated by our finding that infant female rhesus monkeys have higher plasma cortisol concentrations than infant male rhesus monkeys, replicating other studies showing that infant rhesus monkey females have higher stress-induced concentrations of plasma cortisol when compared to infant rhesus monkey males (Capitanio et al., 2005), and studies in humans showing that depressed females have more feminized 2D:4D ratios, when compared to non-depressed females (Smedley et al., 2014; De Kruijff et al., 2016). Perhaps the strongest experimental evidence that testosterone has an organizational effect that attenuates the female response to stress comes from rodent studies. These studies show that when androgens are administered during the organizational phase, the exposed females exhibit an attenuated glucocorticoid response to stress (Seale et al., 2005). In line with this research, individuals with congenital hyperplasia, a condition where the fetus is exposed to high levels of PAE, exhibit dysregulated cortisol biosynthesis as well as a blunted plasma cortisol response to stress (Merke and Bornstein, 2005; Turcu and Auchus, 2016). Furthermore, McHenry et al.’s (2014) comprehensive review of cortisol and the glucocorticoid response to stress and anxiety in animals suggests that, whether exogenous or naturally occurring, PAE decreases anxiety- and depression-like behaviors later in life. Similarly, human males with a more female-like 2D:4D ratio show an increased risk for depression (Bailey and Hurd, 2005), although this appears to have a low effect size and is not always seen in smaller samples (Martin et al., 1999; Li et al., 2019). One possible explanation for the failure of this and some other studies to find a relationship between PAE and cortisol concentrations in males is that males are exposed to substantially higher levels of prenatal androgens than are females (Knickmeyer and Baron-Cohen, 2006), which may reduce interindividual variability (i.e., a ceiling effect), potentially resulting in a failure to detect the same relationship in males. Taken together, these and other findings suggest that a more masculinized 2D:4D ratio is related not only to an attenuated cortisol response in females but also with lower rates of anxiety and depression in both sexes. One possible ramification of these findings and the data presented is that females whose brains are masculinized as a result of higher PAE may be at lower risk for subsequent affective psychopathology.

Consistent with the hypotheses, results showed that females exhibited higher stress-induced plasma cortisol concentrations when compared to males. Studies of sex differences in adult cortisol response have mixed findings, with one meta-analysis concluding that men show higher plasma cortisol concentrations than women (Kudielka and Kirschbaum, 2005). Still, other studies show that sex differences in stress-response plasma cortisol may vary with the type of stressor (Uhart et al., 2006; Goel et al., 2011). Other studies suggest that puberty status may further modulate sex differences in plasma, salivary, and urinary cortisol (Gifford and Reynolds, 2017; Van der Voorn et al., 2017). Human studies investigating sex differences in blood and salivary cortisol in prepubertal children similarly show mixed results (Dahl et al., 1992; Gifford and Reynolds, 2017; Hollanders et al., 2017; Van der Voorn et al., 2017), which may reflect population differences, paradigm, or methodological differences between studies. Perhaps because of the homogeneous rearing and the experimentally manipulated stressors, studies of nonhuman primate infants are more consistent in showing that infant females have higher stress-response plasma cortisol than male infants, particularly when the investigation of sex differences is the primary variable under consideration (Capitanio et al., 2005; Vandeleest et al., 2013). Given the similarity between humans and rhesus monkeys in HPA axis functioning (Sanchez, 2006), this disparity in variability when comparing humans and nonhuman primates may be a consequence of the more controlled early environments, situational testing, and, consequently, increased homogeneity in treatments and early experiences of nonhuman primates although species differences cannot be ruled out. One advantage of the nonhuman primate model is the homogeneous early environment, which increases the capacity to detect effects. While correlation cannot establish causation, one possible explanation is that early PAE has a masculinizing effect on both the 2D:4D ratio and the HPA axis. Future studies should investigate 2D:4D ratio and the HPA axis response perhaps using other measures, such as corticotropin releasing hormone or ACTH, which may give a better estimate of central mechanisms that may be affected by PAE, leading to the sex differences observed.

One possible limitation of these findings is that cortisol concentrations were obtained when subjects were infants, while 2D:4D ratio was measured later in life, spanning a wide range of time (1–17 years later). Nevertheless, studies show that inter-individual differences in stress-induced plasma cortisol concentrations are stable from early in life into adulthood (Higley et al., 1992), and inter-individual differences in 2D:4D finger ratio also appear to be constant across development (Trivers et al., 2006), stabilizing early in life and showing trait-like individual differences across time. For example, Trivers et al. (2006) first measured 2D:4D ratios in *N* = 108 9-year-old Jamaican children and then measured them a second time 4 years later, finding modest inter-individual stability, even though many of the children had gone through puberty between measure one and measure two. Although we did not have repeated 2D:4D measures on all of our subjects, we did repeatedly measure the 2D:4D ratios of a separate representative sample (*N* = 63) across 2 years. Results from bivariate correlations showed a statistically significant positive correlation between the two measurements (*r* = 0.51, *p* < 0.0001), suggesting that, for the present sample, 2D:4D ratio remained stable across time. Subsequent studies are underway to assess the relationship between adult cortisol concentrations and 2D:4D ratios to verify whether the cortisol and 2D:4D relationship is present in adult rhesus monkeys. Studies show that testosterone inhibits cortisol secretion in human adults (for example, see Terburg et al., 2009). It is also of note that, beginning in the first month and ending at the third month of life, there is a postnatal surge in testosterone levels in male, but not female, rhesus monkeys (Robinson and Bridson, 1978; Frawley and Neill, 1979). While this surge is unlikely to have affected cortisol levels in females, it is possible that for the males, measuring cortisol at a later time point may have produced different results, although that remains speculative.

There was a significant sex difference in the left- but not right-hand 2D:4D ratio, with males exhibiting a higher 2D:4D ratio, when compared to females. That the males had a higher 2D:4D ratio, when compared to females, is partially consistent with our earlier study in rhesus monkeys (Baxter et al., 2018), but in that study, the difference was seen in both hands. It is of note that in the previous study, the left hand showed greater sexual dimorphism and the study had a larger sample size, and, as noted in the methods section of this paper, there was a positive correlation between right- and left-hand 2D:4D ratio, suggesting that we may have been underpowered to detect this difference in the right hand.

Finding a relationship between PAE and stress-induced levels of plasma cortisol in female rhesus monkeys is an important first step in investigating the organizational effects of PAE on postnatal HPA axis functioning. Given that much of the early work investigating the masculinizing effect of PAE was performed in rhesus monkeys (Goy et al., 1988), future research should investigate the relationship between PAE and HPA axis functioning through experimental manipulation of PAE in rhesus monkeys, which would lead to evidence for a cause and effect relationship. If such efforts provide further support of the relationship between PAE and HPA axis functioning, 2D:4D ratio may be an important noninvasive biomarker for studies assessing the masculinizing effect of androgens on the HPA axis, as well as studies investigating the early risk for developmental affective disorders, particularly anxiety-related disorders and other HPA axis abnormalities.

## Data Availability Statement

The datasets generated for this study are available on request to the corresponding author.

## Ethics Statement

The animal study was reviewed and approved by the Institutional Animal Care and Use Committee of the University of California, Davis, Davis, CA, United States.

## Author Contributions

EKW, PJ, and JDH contributed to the conception and design of the study, assisted with data analysis and interpretation of findings, and wrote the first draft of the manuscript. EKW, PJ, AB, JPC, and JDH contributed to the acquisition of the data. EKW, PJ, EC, AB, JPC, and JDH wrote sections of the manuscript. All authors contributed to the article and approved the submitted version.

## Conflict of Interest

The authors declare that the research was conducted in the absence of any commercial or financial relationships that could be construed as a potential conflict of interest.
